# Arbitrary Polarization Readout with Dual‐Channel Neuro‐Metasurfaces

**DOI:** 10.1002/advs.202204699

**Published:** 2022-12-15

**Authors:** Zhedong Wang, Chao Qian, Zhixiang Fan, Hongsheng Chen

**Affiliations:** ^1^ ZJU‐UIUC Institute Interdisciplinary Center for Quantum Information State Key Laboratory of Modern Optical Instrumentation Zhejiang University Hangzhou 310027 China; ^2^ ZJU‐Hangzhou Global Science and Technology Innovation Center Key Lab. of Advanced Micro/Nano Electronic Devices & Smart Systems of Zhejiang Zhejiang University Hangzhou 310027 China; ^3^ Jinhua Institute of Zhejiang University Zhejiang University Jinhua 321099 China; ^4^ Shaoxing Institute of Zhejiang University Zhejiang University Shaoxing 312000 China

**Keywords:** neuro‐metasurfaces, polarization detection, polarization atlas, wave independently control, amplitude focus

## Abstract

Polarization, as a vector nature of the electromagnetic wave, plays a fundamental role in optics. Determining the polarization state of light is required by many applications, spanning from remote sensing and material analysis to biology and microscopy. To achieve this goal, conventional methods necessitate cascading of multiple optical components and consequential measurements to estimate the Stokes parameters, rendering the entire optical system bulky, complex, and sensitive. Here a brand‐new strategy is introduced for direct polarization readout based on dual‐channel neuro‐metasurfaces. Neuro‐metasurfaces can independently manipulate two orthogonal linearly‐polarized waves that can synthesize arbitrary polarization waves with a linear combination. By judiciously designing the output focus points, a unique polarization atlas is created that allows one‐to‐one correspondence from intensity ratio to polarization state. To implement this, polarization‐sensitive metasurfaces are designed and the spatial layout is optimized using a diffractive neural network. The feasibility of this strategy is validated by numerical simulation and microwave experiments. These results pave a new avenue in realizing integrated and multifunctional detectors and demonstrate the potential of neuro‐metasurfaces as an add‐on for discomposing and composing spatial basis.

## Graphical abstract

A simple and fast strategy based on multilayer neuron metasurfaces is revealed to determine the lights’ state of polarization. The proposed concept of polarization atlas identifies different polarization states using the amplitude array of designed focus points by neuron metasurfaces. This developed detection method provides promising inroads to downsize the entire detection system, reduce the cost, and simply the procedure.

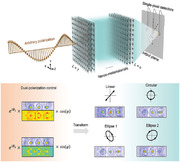

## 1 Introduction

Polarization is a fundamental property of light that characterizes the direction of the vibrating electric field. Together with amplitude, phase, and frequency, they determine how the electromagnetic (EM) wave interacts with matter and thus extract valuable information from the illuminated object.^[^ ^1^ ^]^ Accurately perceiving the state of polarization is a current subject of intense research in a myriad of applications, including optical display, remote sensing, atomic physics, and biology.^[^ ^2^, ^3^, ^4^, ^5^, ^6^, ^7^, ^8^, ^9^ ^]^ For example, polarimetric imaging can reveal valuable information hidden inside the object that is usually unavailable using intensity and spectra‐based detection, such as texture and shape of objects, surface roughness, and constituent material composition.^[^ ^1^, ^10^, ^11^, ^12^ ^]^ Different from intensity and frequency detection, however, polarization determination remains an inherently tricky problem since the phase difference between orthogonal polarization states is completely lost in the detection procedure. As a consequence, early attempts toward this goal have relied on a series of bulky polarizers, wave‐plates, polarization modulators, and consequential measurements to deduce the Stokes parameters that fully describe the state of polarization.^[^ ^13^ ^]^ These methods are indirect and their applications are hampered by the challenges associated with the miniaturization, experimental cost, integration, and exquisite adjustment of high‐quality optical components.

Metasurfaces, as an emerging artificial material, has recently received a great deal of attention.^[^ ^14^ ^]^ They are composed of periodic or quasi‐periodic arrays of subwavelength scatterers, whose EM properties can be manipulated at will through judiciously designing the resonator geometries and orientations. The high degree of freedom in structural design and spatial layout provides a versatile platform for phase, amplitude, and polarization control.^[^ ^15^, ^16^, ^17^, ^18^, ^19^, ^20^, ^21^, ^22^ ^]^ Compared with conventional bulky metamaterials, the distinct advantages of monolithic integration, compact design, and low losses render metasurfaces more attractive in both academia and industry. Thus far, we have witnessed fruitful metasurface applications including wave manipulation, anomalous reflection, invisibility cloak, special beam generation, and so on.^[^ ^16^, ^19^, ^23^, ^24^, ^25^, ^26^, ^27^, ^28^, ^29^, ^30^, ^31^ ^]^

An important characteristic of metasurfaces is associated with polarization sensitivity whose optical responses are different when the polarization of incident wave changes.^[^ ^32^ ^]^ This important characteristic provides a new pathway for determining the polarization state with subtle design and a spectrum of works have validated the feasibility of metasurface‐based polarization detection.^[^ ^33^, ^34^, ^35^, ^36^ ^]^ These works are based on respective intensity measurements in different polarization bases and then retrieve the Stokes vector. Stokes vector, a universal definition of the polarization state, is defined as *S* = [*S*_0_, *S*_1_,*S*_2_,*S*_3_], where *S*_0_ = *I*, *S*_1_ = *I_x_* − *I_y_*, *S*_2_ = *I*_45_ − *I*_−45_, *S*_1_ = *I_R_* − *I_L_* ^[^ ^37^ ^]^. *I* is the total intensity, *I_x_*, *I_y_*, *I*_45_, *I*_−45_, *I_R_*, *I_L_* are the intensity of light in linear polarization bases along the *x*, *y*, +45°, and −45° directions, circular polarization based on right‐hand and left‐hand, respectively. Governed by the Stokes framework, the existing metasurface‐based polarization detection still entails individual metasurface areas and consequential measurements to copy with six polarization components.^[^ ^34^, ^36^ ^]^ In this context, developing compact polarization detection and simple measurement is highly demanded.

In this work, we propose a straightforward and facile method for arbitrary polarization detection based on dual‐channel neuro‐metasurfaces.^[^ ^38^ ^]^ The underlying mechanism is based on a universal physical law that arbitrary polarization can be synthesized by two orthogonal linear polarizations. On this foundation, we design dual‐channel neuro‐metasurfaces that can independently process two linearly‐polarized waves; each linearly‐polarized wave produces three prescribed focus points at the output plane. For an arbitrary polarization wave, we first measure the intensity ratio of three focus points and then determine the polarization state by simply looking up a unique polarization atlas. This “decompose and compose” scheme inherently utilizes the phase and amplitude difference of two orthogonal components to define the polarization state, fundamentally distinctive from conventional works that rely on the Stokes parameter measurement. In the experiment, we design polarization‐sensitive metasurfaces to construct the neuro‐metasurfaces that support diffractive neural networks. Our developed polarization detection provides promising inroads to downsize the entire system, reduce the cost, and simply the complexity, paving the way for fast and simple determination of the light's state of polarization.^[^ ^39^ ^]^

## 2 Results

### 2.1 Working Principle of Polarization Detection with Dual‐Channel Neuro‐Metasurfaces

**Figure 1:**
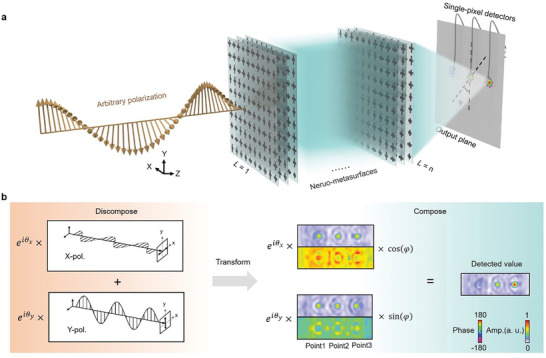
Schematic and working mechanism of the polarization detection enabled by dual‐channel neuro‐metasurfaces. a) Arbitrary polarized wave passing through dual‐channel neuro‐metasurfaces will generate three focus points at the output plane. b) “Discompose and compose” scheme. It is well‐known in physical textbook that arbitrary polarized wave can be synthesized by two orthogonal linearly‐polarized waves (${\bar{E}}_{x}$ and ${\bar{E}}_{y}$). When this arbitrary polarized wave passes through a linear system with little polarization crosstalk, the output is the superposition of the two individual outputs induced by the two orthogonal components, as schematically indicated in (b). Based on this physical principle, we rely on the dual‐channel neuro‐metasurfaces to produce the desired output that enables a one‐to‐one mapping from the detected value to polarization state.

The schematic view of the polarization detection and its “discompose and compose” mechanism are delineated in **Figure** **1**. We consider two basic linearly‐polarized waves (${\bar{E}}_{x}$ and ${\bar{E}}_{y}$) with the phase difference of $\theta_{p}=\theta_{{\bar{E}}_{y}}\;-\theta_{{\bar{E}}_{x}}$ and the amplitude ratio of $R\;=|{\bar{E}}_{y}\left|\;/\right|{\bar{E}}_{x}|$. Classic electromagnetism reveals that two orthogonal linearly‐polarized waves can synthesize arbitrary polarized waves. The mathematical relation is expressed as $\bar{E}\;\left(z\right)={\bar{E}}_{x}\;+R\;{\bar{E}}_{y}=\hat{x}\;e^{ikz}+R\hat{y}e^{ikz+\theta_{p}}$ with the assumption of $|{\bar{E}}_{x}|=\;1$. When a synthetic polarization wave passes through a linear system with negligible polarization crosstalk, the global optical response is the linear superposition of two individual optical responses induced by the two orthogonal waves. In this regard, we introduce dual‐channel neuro‐metasurfaces that can independently deal with two linearly‐polarized waves to produce two sets of predefined output. Therefore, the output of the synthetic polarization wave is correlated to the two sets of output. Reversely, it provides the possibility to determine the polarization state by only measuring the output.

To be specific, we introduce dual‐channel neuro‐metasurfaces to produce three focus points at the output plane (the right of Figure 1a). For ${\bar{E}}_{x}$ polarized wave, the amplitude of three focus points is designed to satisfy: *A*_1*x*_ = *A*_2*x*_ = *A*_3*x*_ , and the corresponding phase satisfies: *p*_1*x*_ − 90° = *p*_2*x*_ = *p*_3*x*_ . For ${\bar{E}}_{y}$ polarized wave, the amplitude of three focus points satisfies: *A*_1*y*_ = *A*_2*y*_ = *A*_3*y*_ , and the corresponding phase satisfies: *p*_1*y*_ = *p*_2*y*_ = *p*_3*y*_ − 90°. Notice that for both ${\bar{E}}_{x}$ and ${\bar{E}}_{y}$ waves, the spatial locations of the three focus points are the same. At the output plane, we use three obliquely‐set single‐pixel probes (with the angle of *φ* in Figure 1a) to detect the focal intensity. According to the vector composition principle, the detected amplitude of the *n*th focus point can be written as

(1) $$\begin{array}{c} A_{n}=A_{nx}\;\times e^{i\theta_{x}}\times e^{i\left(p_{nx}\right)}\times \sin\left(\varphi \right)+A_{ny}\times e^{i\theta_{y}}\times e^{i\left(p_{ny}\right)}\times \cos\left(\varphi \right) \end{array}$$

where *θ*_*x*_ and *θ*_*y*_ represent the initial phase of ${\bar{E}}_{x}$ and ${\bar{E}}_{y}$ component, respectively, and *θ*_*p*_ = *θ*_*y*_ − *θ*_*x*_. One polarization state will induce a unique amplitude array ***A*** = [*A*_1_,*A*_2_,*A*_3_], due to the specific phase and amplitude relationship between ${\bar{E}}_{x}$ and ${\bar{E}}_{y}$ polarized waves. In turn, an inverse mapping *M*: ***A*** → ***Q*** is possible to be built up from the amplitude space ***A*** to the polarization space ***Q*** = [*θ*_p_,*R*] by carefully designing the output attributes.

### 2.2 Structural Design of Polarization‐Sensitive Metasurfaces

**Figure 2:**
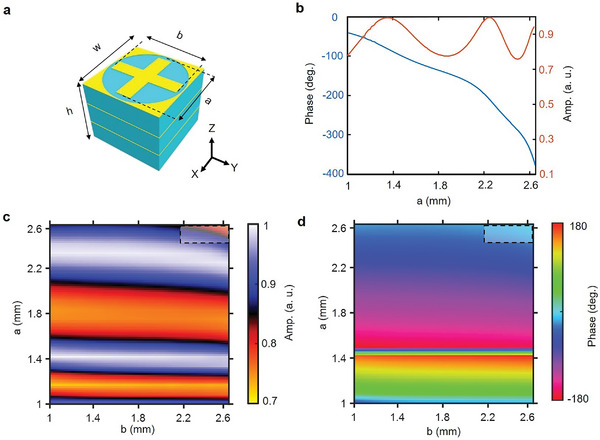
Design of polarization‐sensitive transmissive metasurfaces. a) Illustration of the polarization‐sensitive metasurfaces. The metasurfaces are composed of three substrate layers with the relative permittivity of 2.55, and the other geometrical parameters are: *w* = 6 mm, *h* = 5.01 mm, *a* and *b* vary from 1 mm to 2.65 mm. b) Amplitude and phase variation when *b* is fixed and *a* varies from 1 to 2.65 mm at 16 GHz. c,d) Amplitude and phase variation as the function of *a* and *b*. The transmission amplitude maintains above 0.7 and the phase variation can cover almost 0 to 360°. Dashed line areas are where the polarization coupling starts to appear. It is clear that the independent phase‐control ability holds for the vast majority of area in (c) and (d).

In order to independently control ${\bar{E}}_{x}$ and ${\bar{E}}_{y}$ polarized wave, a high‐transmittance polarization‐sensitive metasurface based on multilayer resonators is designed,^[^ ^40^, ^41^, ^42^, ^43^, ^44^, ^45^ ^]^ as shown in **Figure** **2**a. The metasurfaces are composed of cross‐shaped metal patches and three dielectric substrate layers with a relative permittivity of 2.55 and loss tangent of 0.001. The overall thickness of the metasurfaces is 5.01 mm (among which the thickness of a substrate is 1.6 mm and the metallic thickness is 0.035 mm) and the period is 6 mm. By varying the length of the cross‐shaped metal patches (*a* and *b*) from 1 to 2.65 mm, the transmission phase under ${\bar{E}}_{x}$ and ${\bar{E}}_{y}$ polarized waves can be respectively adjusted in a wide range. The high transmission magnitude and large phase coverage are realized by harnessing the mutual interaction between the metallic structures. As shown in Figure 2b (*a* = 1 mm and *b* ranges from 1 to 2.65 mm), taking ${\bar{E}}_{y}$ polarized wave at 16 GHz as example, the transmission phase covers nearly from 0° to 360° while the transmission amplitude remains a high value (>0.7). Due to the structural symmetry, similar result is also applicable for ${\bar{E}}_{x}$ polarized wave. Figure 2c,d plots the 2D distribution of transmission amplitude and phase variation when *a* and *b* change from 1 to 2.65 mm. It is evident that the length variation of one arm only affects the transmission response of one polarization and almost has no effect on the other polarization. For the tiny region when *a* and *b* are both close to 2.65 mm, the polarization coupling effect starts to appear (dashed line areas in Figure 2c,d); however, this can be easily tackled by adding limitations in the following optimization process to bypass this tiny region.

### 2.3 Training of Dual‐Channel Neuro‐Metasurfaces

**Figure 3:**
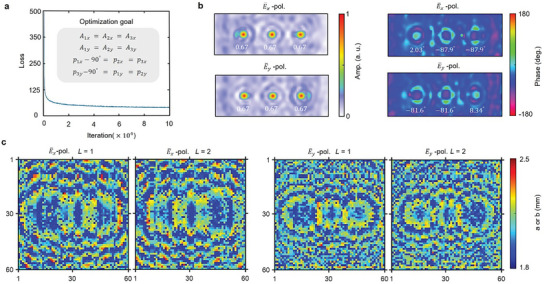
Training of neuro‐metasurfaces polarization detection. a) Loss function curve for the ${\bar{E}}_{y}$ polarization wave. After about 100‐thousand iterations, the loss is converged to the optimization goal. The inset is the detailed goal for two polarization waves. b) Amplitude and phase distribution at the output plane for ${\bar{E}}_{x}$ and ${\bar{E}}_{y}$ polarized waves. The amplitude of all focus points is optimized to 0.67 and their phase difference is optimized to 90°. c,d) Phase distribution (60×60) on two layers for$\;{\bar{E}}_{x}$ and ${\bar{E}}_{y}$ polarized waves.

To design the spatial layout of the metasurfaces, we apply a diffractive neural network.^[^ ^46^, ^47^, ^48^, ^49^ ^]^ The diffractive neural network is physically constructed by multiple layers of diffractive surfaces that work collaboratively to allow powerful wavefront shaping and information communication, which has been widely used in the optical logic operation, image identification, and other inference tasks.^[^ ^46^, ^48^ ^]^ According to the Huygens‐Fresnel principle, each unit cell/neuron can be regarded as a secondary source and connected to others of the following layer through the secondary wave. Here, we consider an optical diffractive network composed of two layers of dual‐channel transmissive metasurfaces, where each unit cell serves as an artificial neuron that is fully connected with all neurons of the following layers through light diffraction. Each layer contains 60 × 60 meta‐atoms and thus the number of connections is 60^4^, providing enough degrees of freedom for manipulating EM waves. During the training process of diffractive neural network, the transmission coefficient of each unit cell/neuron (corresponding to the cross‐structure length of the metasurfaces) is set as a learnable network parameter and iteratively optimized using the error back‐propagation algorithm.^[^ ^47^ ^]^ Training processes are carried out twice for ${\bar{E}}_{x}$ and ${\bar{E}}_{y}$ polarized waves; see the inset of **Figure** **3**a.

Without loss of generality, we choose *f*_0_ = 16 GHz (wavelength *λ*_0_ = 18.75 mm) as the working frequency. In principle, the proposed strategy for polarization detection is applicable for arbitrary frequency as long as the specific metasurfaces are found. For simplicity, the transmission amplitude of each element is assumed to be uniform, and the axial distance between the dual‐channel neuro‐metasurfaces is set to 100 mm. Taking ${\bar{E}}_{y}$ polarized wave as example, we define a loss function as $\left[\sum_{\Omega_{o}}\frac{1}{\left|E\right|}+\sum_{j\;=\;1,2}|f_{A}\left(\Omega_{3}\right)-f_{A}\left(\Omega_{j}\right)|+\sum_{j\;=\;1,2}\left|f_{P}\left(\Omega_{3}\right)\;-\;f_{P}\left(\Omega_{j}\right)\;-\;\frac{\pi }{2}\right|\right]$, where Ω_o_ represents the all focus areas and Ω_*j*_ (*j* = 1, 2, 3) is the area of *j* ^th^ focus point. *f*_A_ is the function to calculate the amplitude on focus points area and *f*_P_ applies a similar calculation for phase. A sufficient number of meta‐atoms ensures fast convergence of the training process, and the loss curve rapidly declines from 500 to around 100 after around 20 thousand iterations (Figure 3a). Totally 100 thousand iterations are operated to ensure the convergence of the neural network. The theoretical results have been illustrated in Figure 3b. For the amplitude part, all focus points reach the same normalized amplitude (*A* = 0.67) for both polarizations. For the phase part, we realize *p*_1*x*_ = 2.03°, *p*_2*x*_ = −87.9°, *p*_3*x*_ = −87.9° for ${\bar{E}}_{x}$ polarized wave and *p*_1*y*_ = −81.6°, *p*_2*y*_ = −81.6°, *p*_3*y*_ = 8.34° for ${\bar{E}}_{y}$ polarized wave. Evidently, *p*_1*x*_ − 90° = *p*_2*x*_ = *p*_3*x*_ and *p*_1*y*_ = *p*_2*y*_ = *p*_3*y*_ − 90° are well satisfied. The ultimate metasurface profiles of two hidden layers are depicted in Figure 3c,d. It is clear that the vast majority of meta‐atoms fall in the region of 1.8–2.2 mm and very few meta‐atoms have two arms’ lengths that are both close to 2.65 mm (to avoid the polarization coupling region).

### 2.4 Numerical Simulation Results

From the analysis, the intensity of the three focus points is induced by the two orthogonally polarized waves based on the vector composition principle. For different *θ*_p_, ${\bar{E}}_{x}$ and ${\bar{E}}_{y}$ components have different phase differences on the single‐pixel probe to ensure each polarization state has unique amplitude values on the three focus points. Based on this principle, the concept of the polarization atlas is proposed. It represents a one‐to‐one mapping relationship from the amplitude space ***A*** to the polarization space ***Q***. Without the calculation of Stokes parameters, one can get rid of the need for complicated experimental procedures, and instead, directly read the polarization state directly from the polarization atlas.

**Figure 4:**
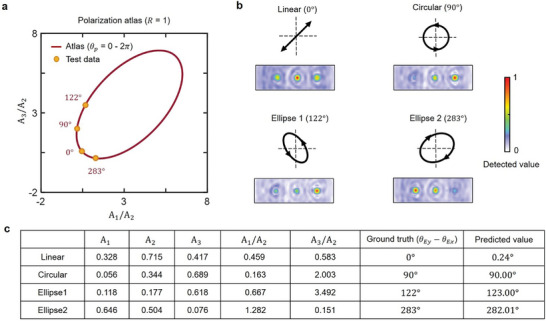
Simulation results. a) Polarization atlas. As an example, we consider the case of *R* = 1 to draw the polarization atlas. Any polarization state corresponds to its unique location in the loop‐curve polarization atlas. The coordinate ($\frac{A_{1}}{A_{2}},\;\frac{A_{3}}{A_{2}}$) is calculated from the amplitude array (*A*_1_, *A*_2_, *A*_3_). b) Simulation results of the polarization detection. The intensity on three focus points for different polarization states (linear, left circular, and ellipse) are quite different, which are used to predict the polarization state by looking up the polarization atlas. c) Detailed values for four randomly‐picked polarization states. The detected results are very coincident with the ground truth.

To verify the feasibility of polarization detection using dual‐channel neuro‐metasurfaces (**Figure** **4**), we first consider the amplitude ratio *R* = 1 (other cases including *R* = 3 and 5 are discussed in Figure S2, Supporting Information) and construct a polarization atlas. Totally 120 simulation results for polarization angle from 0° to 360° are collected firstly and then normalize the amplitude array ***A*** = [*A*_1_, *A*_2_, *A*_3_] to $A^{\prime }=\;\left[\frac{A_{1}}{A_{2}},\;\frac{A_{3}}{A_{2}}\right]$ to reduce the measurement error. As a metaphor, the polarization atlas is more like a map that can guide user to find the destination. In this case, the polarization atlas describes the relationship between the intensity of the three focus points [*A*_1_, *A*_2_, *A*_3_] and the polarization states [*θ*_p_,*R*]. To build up the polarization atlas, we adopt another machine learning model based on nonlinear fitting named generalized regression neural network (GRNN).^[^ ^50^ ^]^ As a member of radial basis neural networks, GRNN has a strong nonlinear approximation ability, making it suitable for this function fitting‐like task. We store the above 120 datasets (amplitude array ***A*** and label *R*) to represents the two spaces ***A*** and ***Q***. And then build up a four‐layer GRNN model, containing an input layer, pattern layer, summation layer, and output layer (Figure S1, Supporting Information), to construct the inverse mapping between ***A*** and ***Q*** so that the polarization atlas can be depicted by GRNN. The accuracy of mapping construction mainly depends on the size of the dataset and the complexity of the inverse problem. Base on the sufficient collected dataset, GRNN can extract the most characteristics of the inverse mapping and restore it, ensuring the prediction error of atlas in a low level. Amplitude array from the focus points is considered as the input and the output is *θ*_p_. The polarization atlas can be exhibited by a curve shown in Figure 4a. Each point on the curve represents a specific polarization state theoretically where the coordinate is the corresponding amplitude array. Therefore, in practice, based on the prepared polarization atlas, we first measure the intensity on three focus points, and directly read the polarization state by table lookup.

We randomly pick several test data including the linear polarization (*θ*_p_ = 0°), right‐hand circular polarization (*θ*_p_ = 90°), right‐hand elliptical polarization (*θ*_p_ = 122°) and left‐had elliptical polarization ( *θ*_*p*_ = 283°)). Then we display the output intensity distribution of the e‐filed in Figure 4b and depict them in Figure 4a (yellow dots) using amplitude information ($\left[\frac{A_{1}}{A_{2}},\;\frac{A_{3}}{A_{2}}\right]$) as coordinate. Apparently, for different polarization states, the three focal‐amplitude are distinctly different, indicating we can determine the polarization state by three focus points directly. The table listed in Figure 4 is the detailed data about prediction results. [*A*_1_,*A*_2_,*A*_3_] in the table is measured by three obliquely‐set probes (see Figure 1a) which is determined by both *x* and *y* polarization components of incoming wave. All prediction errors are within 1°, showing a good agreement with ground truth *θ*_p_.

### 2.5 Experimental Measurement

**Figure 5:**
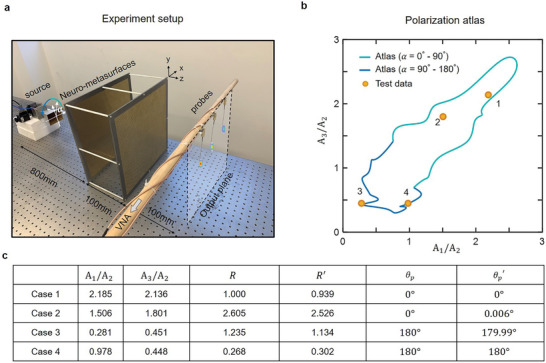
Experiment setup and results. a) Experimental photograph. The neuro‐metasurfaces have a cross section of 400 × 400 mm^2^, and each layer is composed of 60 × 60 elements. Three probes are connected with VNA to collect the amplitude of three focus points. b) Experimental results of the polarization detection. Yellow dots are the measured results and red curve is polarization atlas. c) Detailed values for four tested polarization states. *R*, *θ*_p_ are the ground truth and *R*′, $\theta_{p}^{\prime }$ are the predicted values.

A photograph of the microwave experimental system is provided in **Figure** **5**a. A horn antenna as an excitation source is fixed on a turntable and placed far from the neuro‐metasurfaces (800 mm, ≈ 43*λ*_0_) to guarantee that the measurement is carried out in the far field. The incident wave propagates along the *z*‐axis and vertically impinges on the neuro‐metasurfaces. The neuro‐metasurfaces composed of two transmissive metasurfaces are fabricated by conventional printed circuit board (PCB) technology. The dimensions of a single piece of metasurfaces inclusion are 360 × 360 × 5.25 mm^3^ (60 × 60 unit cells). Incident waves will be converged on the predefined focus points on the output plane (also 100 mm away from the hidden layers). In the measurement, focal intensity on the output plane is collected by three monopole antennas and then transmitted into a vector network analyzer (VNA). To eliminate surrounding interference, the experiment is performed in the microwave anechoic chamber. In the dataset collection, we rotate the antenna from 0° (e‐field vector is along the *x*‐axis) to 180° with the step of 6°, in which the direction of electric field (on the *xoy* section) changes to make *R* and *θ*_p_ change. When the antenna is rotated from 0° to 90° (*α*_1_ region in Figure 5a), *θ*_*p*_ maintains 0° (${\bar{E}}_{x}$ and ${\bar{E}}_{y}$ waves are in phase) but *R* increases from 0. In contrary, when the antenna is rotated from 90° to 180° (*α*_2_ region in Figure 5a), *θ*_p_ changes to 180° and *R* decreases to 0. By utilizing the GRNN model, the polarization atlas that contains all the polarization states for the rotating antenna is drawn by a blue looped curve (Figure 5b). Arbitrary polarization can be directly readout from the polarization atlas by matching the point (*A*_1_/*A*_2_, *A*_3_/*A*_2_), where *A*_1_, *A*_2_, *A*_3_ are the intensity of three focus points. GRNN features to construct the inverse mapping mainly based on the measured dataset, which means the interference such as noise has been taken into account automatically in GRNN. Thus, although the polarization atlas is slightly deformed due to the measurement error and environmental noise, the prediction performance is not disturbed severely. To validate the polarization atlas, we randomly pick up several polarization states with different *R* and *θ*_p_ (yellow dots in Figure 5b). The detailed prediction results are listed in Figure 5c, where *R*, *θ*_p_ are the ground truth and *R*′, $\theta_{p}^{\prime }$ are the prediction values. From the table, it is clear that the prediction error between *R* and *R*′ is small than 0.1° and $\theta_{p}^{\prime }$ is also consistent with *θ*_p_, suggesting that the polarization atlas can determine the polarization states with high accuracy. The underlying reason that causes detection error in experiment results in case 1 or case 2 may be due to the lack of the number of data set when *R* becomes large. In addition, the measurement error arising from experimental settings also disturbs the results. These can be solved by enlarging data set and improving experimental settings.

## 3 Conclusion

In summary, a direct polarization readout method by dual‐channel neuro‐metasurfaces is proposed and experimentally demonstrated in the microwave. This strategy is radically different from previous works that need to completely measure the Stokes parameters. Alternatively, we only measure the intensity of three focus points and then obtain the polarization state by simply looking up a polarization atlas. The underlying “discompose and compose” scheme based on dual‐channel neuro‐metasurfaces holds great potential to not only make the entire system compact but also promise to reshape the landscapes of multifunctional wave manipulation. Furthermore, this approach paves an innovative way for the next generation of compact polarization detection and inspires other massive emergent detections, such as chiral molecule and material composition.

## 4 Experimental Section

### Optimization of Dual‐Channel Neuro‐Metasurfaces

The neuro‐metasurfaces were optimized on a computer (NVIDIA Tesla A100 and Intel(R) Xeon(R) CPU i9‐1085K with 128 GB RAM). The Python version was 3.7.0. The neuro‐metasurfaces consisted of two transmissive metasurfaces, each of which had 60×60 elements. For two polarization states, the metasurface profile was separately optimized based on a diffractive neural network, taking around 1 hour for each case.

### Experiment Setup

The microwave experiment was operated in an anechoic chamber. A horn antenna was set normally toward neuro‐metasurfaces as an excitation source. The gain of the horn antenna was 20 dB and the working frequency was from 12 to 18 GHz. A near‐field 3D auto‐scanning system was used for measurements. Vector network analyzer (VNA) was connected with small monopoles probe used to scan the local e‐field intensity. In the measurement, the transmitting antenna was connected to Port 1 and the monopole probes to other ports, and then the near‐field scanning system automatically recorded the electric distribution at the output layer.

## Conflict of Interest

The authors declare no conflict of interest.

## Author Contributions

C.Q., Z.W., and H.C. conceived the idea. Z.W. conducted the numerical simulations and experiments. Z.F. helped the training of neural network. Z.W. and C. Q. contributed to the writing of the manuscript. C.Q. and H.C. supervised the project.

## Supporting information

Supporting Information

## Data Availability

The data that support the findings of this study are available from the corresponding author upon reasonable request.
