# Supplementary material for: Arbitrary Polarization Readout with Dual‐Channel Neuro‐Metasurfaces
Source: Adv Sci (Weinh). 2022 Dec 15;10(5):2204699. doi: 10.1002/advs.202204699 (PMC9929113; doi:10.1002/advs.202204699)
Supplement: Supplementary file 1 — Supporting Information [file ADVS-10-2204699-s001.pdf]

ADVANCED SCIENCE
Open Access

Supporting Information

for *Adv. Sci.*, DOI 10.1002/advs.202204699

Arbitrary Polarization Readout with Dual-Channel Neuro-Metasurfaces

Zhedong Wang, Chao Qian, Zhixiang Fan and Hongsheng Chen**

Supplementary information for

Arbitrary polarization readout with dual-channel neuro-metasurfaces

Zhedong Wang[1,2,3], Chao Qian[1,2,3,*], Zhixiang Fan, and Hongsheng Chen[1,2,3,*]

[1] *ZJU-UIUC Institute, Interdisciplinary Center for Quantum Information, State Key Laboratory of Modern Optical Instrumentation, Zhejiang University, Hangzhou 310027, China.*

[2] *ZJU-Hangzhou Global Science and Technology Innovation Center, Key Lab. of Advanced Micro/Nano Electronic Devices & Smart Systems of Zhejiang, Zhejiang University, Hangzhou 310027, China.*

[3] *Jinhua Institute of Zhejiang University, Zhejiang University, Jinhua 321099, China.*

[*] *Corresponding author: chaoq@intl.zju.edu.cn (C. Qian); hansomchen@zju.edu.cn (H. Chen)*

Supplementary Note 1: Drawing the polarization atlas using generalized regression neural network

To build up the arbitrary polarization atlas, we need to restore the unique inverse mapping $M\!:\boldsymbol{A} \to \boldsymbol{Q}$ which is from the amplitude space $\{\boldsymbol{A} = [A_1, A_2, A_3]\}$ to the polarization space $\{\boldsymbol{Q} = [\theta_p\ , R]\}$. To this end, we adopt a GRNN (Generalized regression neural network) machine learning model.

As shown in Fig. S1, GRNN contains four layers, including an input layer, a pattern layer, a summation layer, and an output layer. Considering a case that an inverse mapping $M\!:\boldsymbol{A}^K \to \boldsymbol{Q}^M$ from the amplitude space $\{\boldsymbol{A} = [\ A_1, A_2, A_3\ ]\}$ to the source space $\{\boldsymbol{Q} = [\theta_p\ , R]\}$, wherein K is the number of inputs (K = 3) and M is the number of parameters need to be predicted (M = 2). To reduce the measurement error, we set $\boldsymbol{A_i} = [\frac{A_1}{A_2}, \frac{A_3}{A_2}]$ as an input vector and fed to the input layer. The pattern layer will calculate the value of Gaussian function between input data and dataset $\boldsymbol{A}_d$. Then, the output from the pattern layer is fully connected with the summation layer with connection weights $\boldsymbol{L}_g$ (except for the red node with connection weights L_r shown in Fig. S1, the red node means the sum of the pattern layer outputs) calculates the weighted sum of the pattern layer outputs by using y_{ij} as the weighting factor, where y_{ij} is the ith element in the jth label data array. Therefore, the output from summation layer can be written as

$$L_r = \sum_{j=1}^{N} e^{-\frac{\|\boldsymbol{A_i} - \boldsymbol{A}_d(j)\|}{2\delta^2}} \tag{S1}$$

where δ is GRNN's hyperparameter controlling the effect of the bias functions and N is the number

of labeled data set. L_r is the red node in the summation layer, and other green nodes' output are written as,

$$\boldsymbol{L_g} = \sum_{j=1}^{N} y_{ij} e^{-\frac{\|a(u)-a(j)\|}{2\delta^2}} \quad (2)$$

wherein $i = 1, \ldots, M$. Therefore, the prediction result is

$$\boldsymbol{S}(out) = \frac{\boldsymbol{L_g}}{L_r} \quad (3)$$

For polarization deteciton, the output is $\boldsymbol{Q} = [\theta_p, R]$. When a sufficient number of datasets are collected, the amplitude vector sampled by the probes can be processed by the GRNN to predict arbitrary polarization state $\boldsymbol{Q} = [\theta_p, R]$ with high fidelity.

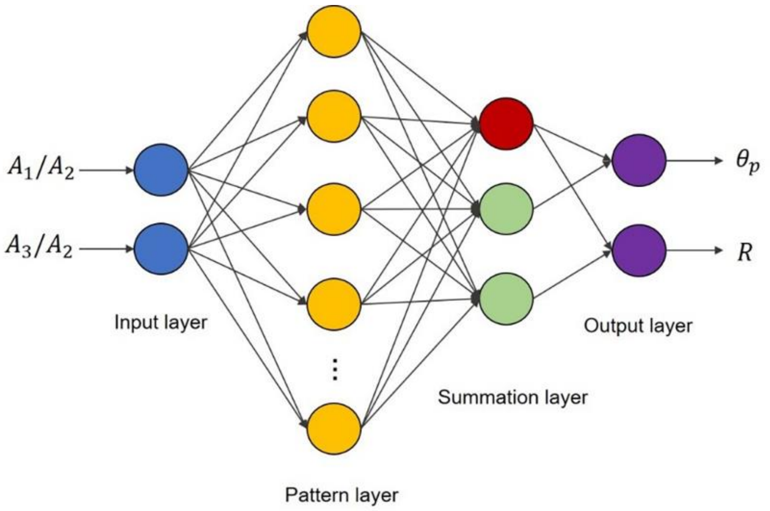

FIG. S1 | The structure of GRNN. GRNN generally contains four layers, one input layer, pattern layer, summation layer, and output layer. The input layer has the nodes same as the input vector and the pattern layer has the node number same as the size of dataset. The pattern layer operates the Gaussian function between input data and dataset and output to the summation layer does a summation. The output layer divides data from the red node by data from the green nodes on summation layer and outputs the final results.

Supplementary Note 2: Examples of arbitrary polarization detection

An incident wave with an arbitrary polarization state can be described by the polarization angle θ_p and amplitude ratio R, as discussed in the main text. We set R = 3 and 5 as example and consider θ_p varies from 0° to 360°. When R = 3, in the dataset collection, totally 120 simulation results are collected firstly and then normalize the amplitude array $\boldsymbol{A} = [A_1, A_2, A_3]$ to $\boldsymbol{A'} = [\frac{A_1}{A_2}, \frac{A_3}{A_2}]$. To

build up the polarization atlas, we store the above datasets (amplitude array $\boldsymbol{A}$ and label R) to represents the two spaces $\boldsymbol{A}$ and $\boldsymbol{Q}$ ($\boldsymbol{Q} = [\theta_p]$ in this case), then build up a four-layer GRNN model (the hyperparameter δ = 0.01) to retrieve the inverse mapping between $\boldsymbol{A}$ and $\boldsymbol{Q}$ so that the polarization atlas can be depicted by GRNN. The polarization atlas can be exhibited by a red curve shown in Fig. S2a. Each point on the curve represents a specific polarization state theoretically where the coordinate is the corresponding amplitude array. Several θ_p are randomly picked as test data (yellow dots in Fig. S2a). Figure S2b shows the intensity distribution of e-field on focus areas where θ_p = 36°, 109°, 182°, 255°. It is evident that for different polarizations, the intensity for three focus points is distinctly different which means polarization states can be inferred from focal-intensity directly. The table in Fig. S2a lists the detailed values of the prediction. The results show that the prediction errors between the ground truth θ_p and predicted θ_p are within 1°. The case of $R = 5$ is demonstrated with the same procedure, and the predicted results are shown in Fig. S2b, where the predicted θ_p also show a good agreement with the ground truth θ_p.

When R and θ_p both vary, the polarization atlas is presented in three dimensions, in which coordinate (x, y, z) corresponds to $(\frac{A_1}{A_2}, \frac{A_3}{A_2}, R)$. The above R-constant situations are slices of this 3-D closed surface. For $\boldsymbol{Q} = [\theta_p, R]$ prediction, we set R = 1, 2, 3, 5 and θ_p ranges from 0° to 360° as examples (Fig. S3). Totally 480 data is collected (120 for each R) to represent $\boldsymbol{A}$ and $\boldsymbol{Q}$ spaces. We build the polarization atlas by four-layer GRNN model (δ = 0.01) and display the polarization atlas in two-dimensional using curves with different shades in red. From the figure, it is found that each polarization state $\boldsymbol{Q}$ has a unique position, meaning the one-to-one mapping relation from $\boldsymbol{A}$ to $\boldsymbol{Q}$ still holds when both θ_p and R change. The table in Fig. S3 shows the prediction values in detail. R, θ_p are ground truth and R', $\theta_p{}'$ represent prediction values. Several θ_p are randomly picked as test data to validate the polarization atlas (yellow dots in Fig. S3). From the table, the prediction error between R and R' is all almost 0 and the error between θ_p and $\theta_p{}'$ are within 1°, meaning that prediction values match with the ground truth. These results show the strategy based on the "decompose and compose" scheme can outstrip conventional methods in terms of the simplicity and accuracy.

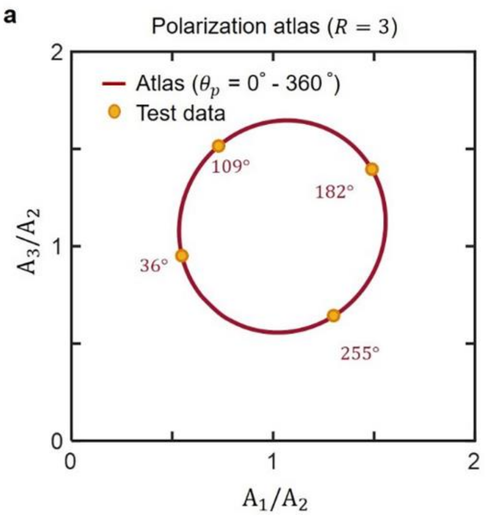

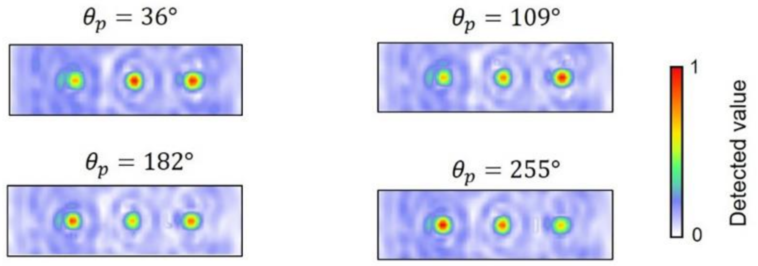

Ground truth θ_p	36°	109°	182°	255°
Predicted value θ_p	35.99°	108.27°	182.93°	255.01°

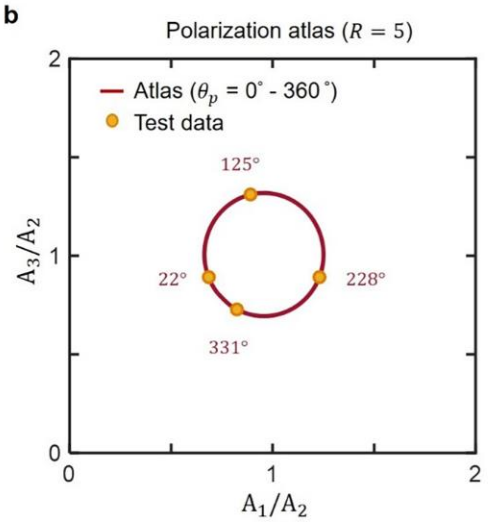

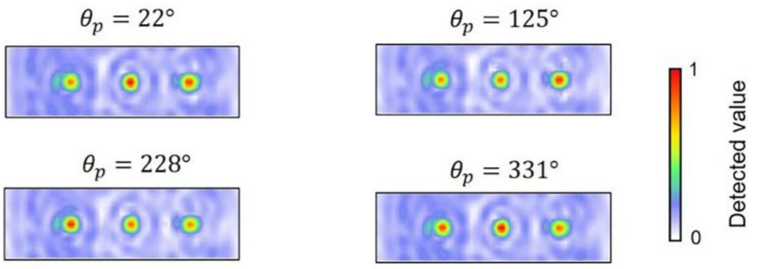

Ground truth θ_p	22°	125°	228°	331°
Predicted value θ_p	21.98°	125.07°	228.02°	331.15°

FIG. S2 | Demonstration of polarization atlas under arbitrary polarization angle. a and **b** are the results of $R = 3$ and 5, respectively. Several polarization states are picked up randomly (yellow dots) and predicted using a predetermined polarization atlas (red curves). Figures on the right top are the focal intensity of the three focus points under different polarization angles. The tables show the ground truths and the predicted values in detail for four different cases.

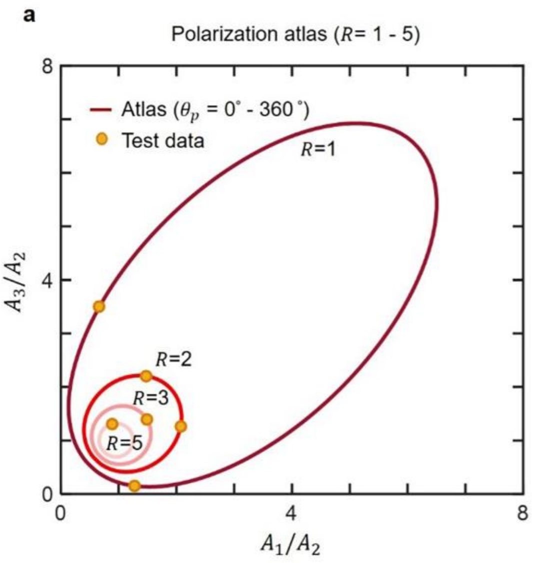

b

	A_1/A_2	A_3/A_2	R	R'	θ_p	$\theta_p{}'$
Case 1	0.667	3.497	1	1	122°	123.00°
Case 2	1.281	0.151	1	1	283°	282.00°
Case 3	2.074	1.269	2	2	203°	204.00°
Case 4	1.481	2.203	2	2	154°	153.00°
Case 5	1.489	1.395	3	3	182°	182.93°
Case 6	0.891	1.311	5	5	125°	125.07°

FIG. S3 | Demonstration of polarization atlas under arbitrary polarization. a, Polarization atlas with different R and θ_p. Several polarization states are picked up randomly and predicted (yellow dots). **b**, Detailed values for the tested polarization states.
